# Internal consistency reliability is a poor predictor of responsiveness

**DOI:** 10.1186/1477-7525-3-33

**Published:** 2005-05-09

**Authors:** Milo A Puhan, Dianne Bryant, Gordon H Guyatt, Diane Heels-Ansdell, Holger J Schünemann

**Affiliations:** 1 Horten Centre, University Hospital, Postfach Nord, 8091 Zurich, Switzerland; 2 Department of Clinical Epidemiology and Biostatistics, McMaster University, 1200 Main St. W., Health Sciences Centre, Room 2C12, Hamilton, Ontario, L8N 3Z5 Canada; 3 Department of Medicine, McMaster University, Health Sciences Centre, Hamilton, Ontario, L8N 3Z5 Canada

## Abstract

**Background:**

Whether responsiveness represents a measurement property of health-related quality of life (HRQL) instruments that is distinct from reliability and validity is an issue of debate. We addressed the claims of a recent study, which suggested that investigators could rely on internal consistency to reflect instrument responsiveness.

**Methods:**

516 patients with chronic obstructive pulmonary disease or knee injury participating in four longitudinal studies completed generic and disease-specific HRQL questionnaires before and after an intervention that impacted on HRQL. We used Pearson correlation coefficients and linear regression to assess the relationship between internal consistency reliability (expressed as Cronbach's alpha), instrument type (generic and disease-specific) and responsiveness (expressed as the standardised response mean, SRM).

**Results:**

Mean Cronbach's alpha was 0.83 (SD 0.08) and mean SRM was 0.59 (SD 0.33). The correlation between Cronbach's alpha and SRMs was 0.10 (95% CI -0.12 to 0.32) across all studies. Cronbach's alpha alone did not explain variability in SRMs (p = 0.59, r^2 ^= 0.01) whereas the type of instrument was a strong predictor of the SRM (p = 0.012, r^2 ^= 0.37). In multivariable models applied to individual studies Cronbach's alpha consistently failed to predict SRMs (regression coefficients between -0.45 and 1.58, p-values between 0.15 and 0.98) whereas the type of instrument did predict SRMs (regression coefficients between -0.25 to -0.59, p-values between <0.01 and 0.05).

**Conclusion:**

Investigators must look to data other than internal consistency reliability to select a responsive instrument for use as an outcome in clinical trials.

## Background

Health-related quality of life (HRQL) instruments should demonstrate adequate test-retest reliability, cross-sectional and longitudinal validity before investigators use them to assess outcomes in research studies. Whether responsiveness, the ability of an instrument to detect change in HRQL when change occurs, is a measurement property distinct from reliability and validity remains, however, controversial [1-4].

Lindeboom et al. purportedly tested the assumption that responsiveness is not a distinct measurement property, but is embodied in internal consistency reliability [5]. To investigate their hypothesis, the authors removed the item contributing most to internal consistency (as determined using Cronbach's alpha) in a step-wise fashion from the physical component of the Sickness Impact profile, the Barthel activities of daily living scale and the psychosocial domain of the Graves' ophthalmology quality of life instrument using data from three previous studies. Following each step-wise removal, they recalculated Cronbach's alpha and the standardised response means (SRM, change score divided by standard deviation of change score) of the remaining items. They then assessed the correlation of these new Cronbach's alphas with the new SRMs and observed strong associations (Spearman rank correlation coefficients between 0.90 and 1.00). They concluded that internal consistency reliability adequately reflects an instrument's responsiveness and that investigators can use the two entities interchangeably.

The first conceptual problem with the approach Lindeboom et al. chose is that they looked at the correlation of internal consistency reliability and responsiveness within single studies and instruments only. However, this approach does not take into account that responsiveness depends on the type of an intervention while internal consistency reliability does not. Most HRQL measures may be very reliable, but internal consistency reliability has nothing to do with the therapy that is producing the change. In contrast, if an intervention targets aspects of HRQL that are specifically covered by a disease-specific instrument, for example, responsiveness is likely to be high. If the effect of another intervention targeting aspects other than those covered by the instrument, responsiveness will be lower. Thus the within study approach does not take into account that responsiveness is not a fixed measurement property.

Another important issue to consider is the influence of other determinants of an instrument's responsiveness such as the type of instrument, generic or disease-specific. There is ample evidence that responsiveness depends on the type of instrument. [6-9] Lindeboom's within instrument approach does not take into account this issue.

Finally, if the within instrument approach with step-wise deconstruction of domains is used, one would expect step-wise decreases of internal consistency reliability, responsiveness and other measurement properties such as cross-sectional validity for the following reasons. Internal consistency reliability is reduced when the items contributing most to internal consistency reliability are removed because the error term in the denominator increases. For the same reason, responsiveness deteriorates if the number of items is decreased[10]. Thus it is likely to see a parallel decline of internal consistency reliability and responsiveness even if there is no relationship between these two measurement properties. Indeed using Lindeboom's approach one would expect high correlations between internal consistency reliability and other measurement properties such as cross-sectional validity and could consequently conclude that they are all embodied in internal consistency reliability. The assessment of the relationship between internal consistency reliability and responsiveness should include entire domains, as they were developed, validated and used in research.

Having considered the methodological challenges and constraints above, we analysed the relationship between internal consistency reliability and responsiveness of entire domains across different instruments and studies using data from several of our previous studies.

## Methods

### Studies

A priori we defined the following eligibility criteria to ensure an unbiased selection of datasets as possible and to ensure that it was theoretically possible to detect a correlation between internal consistency reliability and responsiveness if one existed. We applied the following criteria:

1. Studies must have longitudinal follow-up with a baseline assessment and at least one follow-up assessment completed by the CLARITY research group (McMaster University, Hamilton, Ontario, Canada) within the last five years.

2. Studies must have investigated an intervention of established effectiveness that induces changes in HRQL.

3. Studies must include ≥ 2 multi-item HRQL instruments that allow calculation of Cronbach's alpha and instruments within a study must have different degrees of responsiveness (e.g. generic versus disease-specific) to ensure variability in responsiveness. We expected variability in Cronbach's alpha to be limited to values ≥ 0.60 because only those are generally accepted to represent sufficient internal consistency reliability [3].

### Statistical analysis

We calculated Cronbach's alpha using baseline scores for each domain of each HRQL instrument or for the total instrument if domains did not exist. Similarly, for each domain or for a total score we calculated SRMs (change score divided by standard deviation of change score).

We calculated the correlation between Cronbach's alpha and the corresponding SRM using Pearson correlation coefficients across all studies and for each study separately. We then built linear regression models with the SRM as the dependent variable and Cronbach's alpha as the independent variable. Since the type of instrument (generic or disease-specific) affects the SRM [6-9], we introduced the type of instrument as a covariate into the regression models. For all regression models, we adjusted for possible clustering for data originating from the same group of patients (for example, patients from one study providing data for eight domains of the Short-Form Survey 36) by using the cluster function of STATA. We performed all statistical analysis with STATA for Windows version 8.2 (StataCorp, College Station, Texas, USA).

## Results

### Eligible Studies

The following four studies met the eligibility criteria:

#### Study 1 [11]

This prospective study measured HRQL in 85 patients with chronic obstructive pulmonary disease (COPD) before and after participation in Canadian inpatient respiratory rehabilitation programs similar to many inpatient programs worldwide [12]. All patients completed the interviewer-administered Chronic Respiratory Questionnaire (CRQ) including individualised and standardised dyspnea questions. In addition, patients completed the St. Georges Respiratory Questionnaire (SGRQ) and the Short-From Survey 36 (SF-36) [13] at the beginning and end of the rehabilitation program.

#### Study 2 [14]

This was a prospective randomised study of 177 patients with COPD before and after respiratory rehabilitation in Canada and the United States. We randomised patients to complete either the interviewer or self-administered CRQ [11,15]. All patients answered the individualised and standardised dyspnea questions of the CRQ. Patients also completed the SGRQ and the SF-36 at the beginning and end of the rehabilitation program.

#### Study 3 [16,17]

This prospective study enrolled 71 patients with COPD following a respiratory rehabilitation program at four cites in Switzerland, Germany and Austria. We also randomised patients to complete either the interviewer or self-administered CRQ as in study 2 [11,15] and all patients answered the individualised and standardised dyspnea questions of the CRQ. Patients also completed the SF-36 [18] at the beginning and end of the rehabilitation program.

#### Study 4 [19]

This prospective study enrolled patients undergoing anterior crucial ligament reconstruction (study 4a, n = 66) and knee arthroscopy (study 4b, n = 117) to determine their ability to recall pre-operative quality of life and functional status. Patients completed the disease-specific Anterior Crucial Ligament Quality Of Life questionnaire (ACL-QOL) [20] (study 4a) or the Western Ontario Meniscal Evaluation Tool (WOMET) [21] (study 4b) as well as the International Knee Documentation Committee (IKDC) Subjective Form [22], the Knee Injury and Osteoarthritis Outcome Score (KOOS) [23] and the SF-36 pre- and one year post-operatively.

### Relationship between internal consistency reliability and responsiveness

Tables 1 and 2 show the reliability coefficients and standardised response mean for each study and instrument. The mean Cronbach's alpha across all studies was 0.83 (SD 0.08, range 0.61 to 0.97) and the mean standardised response mean was 0.59 (SD 0.33, range -0.08 to 1.45).

**Table 1:** Internal consistency reliability and responsiveness. Studies 1 and 2

**Study**	**Instrument and domain**	**Internal consistency reliability^¶^**	**Standardised response mean^#^**
**Study 1**	**CRQ-IA**		
	Dyspnea individualised	0.78	1.16
	Dyspnea standardised	0.82	0.81
	Fatigue	0.86	0.85
	Emotional function	0.88	0.76
	Mastery	0.75	0.95
	**SGRQ**		
	Symptoms	0.63	0.27
	Activities	0.75	0.49
	Impact	0.73	0.69
	Total	0.84	0.66
	**SF-36**		
	Physical Functioning	0.80	0.83
	Role Physical	0.77	0.54
	Bodily Pain	0.93	0.29
	General Health	0.64	0.13
	Vitality	0.80	0.65
	Social Functioning	0.74	0.63
	Role Emotional	0.84	0.50
	Mental Health	0.84	0.41
**Study 2**	**CRQ-IA**		
	Dyspnea individualised	0.77	0.66
	Dyspnea standardised	0.86	0.50
	Fatigue	0.80	0.25
	Emotional function	0.90	0.24
	Mastery	0.92	0.38
	**CRQ-SA**		
	Dyspnea individualised	0.83	0.84
	Dyspnea standardised	0.86	0.69
	Fatigue	0.84	0.60
	Emotional function	0.89	0.56
	Mastery	0.87	0.70
	**SGRQ**		
	Symptoms	0.72	0.34
	Activities	0.81	0.33
	Impact	0.80	0.46
	Total	0.88	0.51
	**SF-36**		
	Physical Functioning	0.87	0.31
	Role Physical	0.91	0.19
	Bodily Pain	0.83	0.22
	General Health	0.76	0.22
	Vitality	0.86	0.32
	Social Functioning	0.90	0.21
	Role Emotional	0.92	0.07
	Mental Health	0.87	0.15

¶Cronbach's alpha; # Change score (follow-up minus baseline) / standard deviation of change score; * Pearson correlation coefficient; CRQ-IA interviewer-administered CRQ; CRQ-SA self-administered CRQ

**Table 2:** Internal consistency reliability and responsiveness. Studies 3 and 4

**Study**	**Instrument and domain**	**Internal consistency reliability^¶^**	**Standardised response mean^#^**
**Study 3**	**CRQ-IA**		
	Dyspnea	0.73	0.94
	Fatigue	0.81	0.96
	Emotional function	0.77	1.35
	Mastery	0.76	1.09
	**CRQ-SA**		
	Dyspnea	0.78	0.86
	Fatigue	0.83	1.38
	Emotional function	0.89	1.00
	Mastery	0.86	0.86
	**SF-36**		
	Physical composite score	0.61	0.59
	Mental composite score	0.70	0.48
**Study 4a**	**ACL-QOL**	0.96	1.45
	**IKDC**	0.86	1.17
	**KOOS**		
	Symptoms	0.74	0.64
	Pain	0.89	0.74
	Function	0.96	0.59
	Sports	0.92	0.83
	QOL	0.88	1.15
	**SF-36**		
	Physical Function	0.90	0.64
	Role Physical	0.79	0.67
	Bodily Pain	0.80	0.52
	General Health	0.72	0.11
	Vitality	0.87	0.44
	Social Functioning	0.81	0.50
	Role Emotional	0.82	0.33
	Mental Health	0.76	0.34
**Study 4b**	**WOMET**	0.97	0.88
	**IKDC**	0.90	0.85
	**KOOS**		
	Symptoms	0.82	0.85
	Pain	0.93	0.95
	Function	0.97	0.82
	Sports	0.94	0.66
	QOL	0.90	0.71
	**SF-36**		
	Physical Function	0.93	0.59
	Role Physical	0.93	0.40
	Bodily Pain	0.88	0.60
	General Health	0.85	-0.08
	Vitality	0.86	0.08
	Social Functioning	0.84	0.25
	Role Emotional	0.88	0.12
	Mental Health	0.79	0.21

¶Cronbach's alpha

# Change score (follow-up minus baseline) / standard deviation of change score

* Pearson correlation coefficient

**Figure 1:**
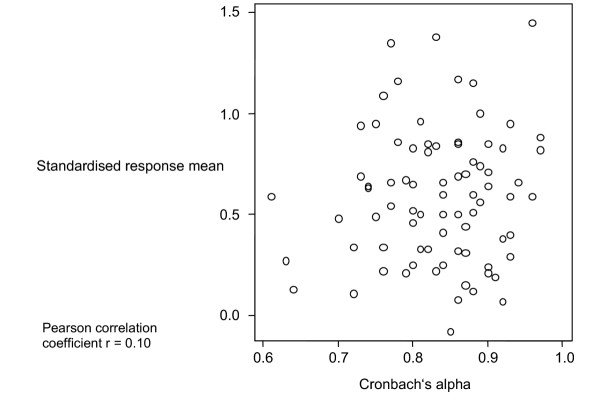
**Relationship between internal consistency reliability and responsiveness, all studies **Relationship between Cronbach's alpha and standardised response mean for 79 domains or total scores of health-related quality of life instruments and symptoms scales. The data come from four studies including 333 patients with chronic obstructive pulmonary disease following a pulmonary rehabilitation and 183 patients with knee injury undergoing anterior crucial ligament reconstruction or knee arthroscopy.

**Figure 2:**
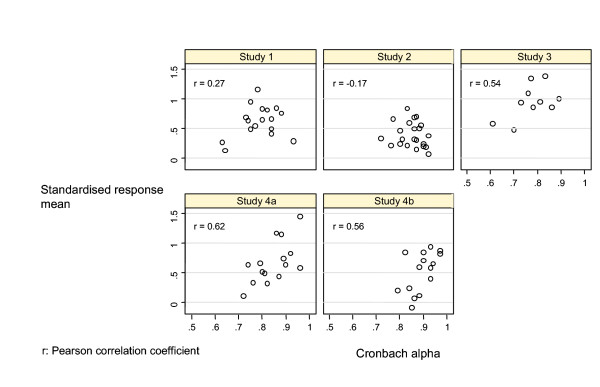
Relationship between internal consistency reliability and responsiveness, per study.

Figure 1 shows the relationship between Cronbach's alpha and SRM across all studies. The correlation coefficient was 0.10 (95% CI -0.12 to 0.32). When we analysed each study separately, correlation coefficients ranged from -0.17 to 0.62 (Figure 2).

Table 3 shows the regression equations to predict the SRM from Cronbach's alpha. In an analysis of all studies including internal consistency reliability as the sole independent variable did not predict responsiveness (p = 0.59, r^2 ^= 0.01). In contrast, an analysis that included the type of instrument showed that the generic versus specific categorisation predicted responsiveness (p = 0.01, r^2 ^= 0.37). Analysing the studies separately showed similar results (Figure 2). Only in study 4 was Cronbach's alpha a significant predictor in unadjusted analyses. Even in this case, when we introduced the type of instrument into the model, Cronbach's alpha was no longer a significant predictor.

**Table 3:** Prediction of responsiveness from internal consistency reliability

**Study**	**Dependent variable**	**Independent variables**	**Constant**	**Regression coefficient (p-value)**	**R^2^**
**All studies**	SRM	Cronbach's alpha	0.25	0.41 (0.59)	0.01
	SRM	Cronbach's alpha	0.66	0.13 (0.83)	0.37
		Type of instrument*		-0.40 (0.01)	
**Study 1**	SRM	Cronbach's alpha	-0.09	0.90 (0.30)	0.07
	SRM	Cronbach's alpha	-0.07	1.04 (0.18)	0.32
		Type of instrument*		-0.25 (0.04)	
**Study 2**	SRM	Cronbach's alpha	0.94	-0.64 (0.46)	0.03
	SRM	Cronbach's alpha	0.52	-0.02 (0.98)	0.48
		Type of instrument*		-0.29 (<0.01)	
**Study 3**	SRM	Cronbach's alpha	-0.51	1.88 (0.11)	0.29
	SRM	Cronbach's alpha	1.42	-0.45 (0.74)	0.60
		Type of instrument*		-0.59 (0.05)	
**Study 4a**	SRM	Cronbach's alpha	-1.81	2.94 (0.013)	0.39
	SRM	Cronbach's alpha	-0.46	1.58 (0.15)	0.60
		Type of instrument*		-0.37 (0.03)	
**Study 4b**	SRM	Cronbach's alpha	-2.61	3.52 (0.03)	0.32
	SRM	Cronbach's alpha	-0.43	1.36(0.22)	0.74
		Type of instrument*		-0.48 (<0.01)	

SRM: standardised response mean

* Disease-specific or generic

## Discussion

We assessed the relationship between internal consistency reliability and responsiveness and found no evidence to support the claim that investigators can use them interchangeably. In general, internal consistency reliability is a poor predictor of responsiveness. Consistent with previous findings [6], we showed that in contrast to Cronbach's alpha, a significant predictor of responsiveness is whether the instrument is a generic or a disease-specific HRQL instrument.

Our findings contradict those presented by Lindeboom et al. We suspect that these differences are largely due to differences in conceptual and, thus, statistical approaches. In particular, Lindeboom's within instrument and within study approach fails to take into account that responsiveness depends on the type of instrument and on the intervention that produces change in HRQL. In our analyses, we evaluated the relationship between internal consistency reliability and responsiveness across instruments and studies.

One might argue that our failure to demonstrate a relationship between Cronbach's alpha and the SRM results from the limited variability in Cronbach's alpha across the instruments and their domains. Indeed, this limited variability in part explains the lack of relationship. Nevertheless, when choosing instruments for clinical trials, investigators will face Cronbach's alpha coefficients such as those shown in Table 1 and 2. If they rely on these results to predict responsiveness, they will be misled. In particular, some domains with very high Cronbach's alpha coefficients (SF-36 bodily pain, 0.93; CRQ IA emotional function 0.90) had low responsiveness (SRMs of 0.29 and 0.24, respectively).

Strengths of our study include the definition of a priori criteria to ensure an unbiased selection of studies that ensure large variability responsiveness creating the greatest potential to detect a relationship if one existed. Furthermore, the inclusion of very different patient populations (chronic lung disease and knee pathology) and the consistency of results across these studies and populations enhances the generalizability of our study. Replication in other populations would further strengthen our conclusions.

## Conclusion

Our study demonstrates that internal consistency reliability is a poor predictor of responsiveness and that both conceptual and statistical evidence exists to support the argument that they are distinct measurement properties of evaluative instruments.

## Authors' contributions

MAP, DB, GHG and HJS designed the study and wrote the study protocol. MAP, GHG, HJS and DB collected the data. MAP, DB and DHA performed the statistical analysis. MAP drafted and DB, GHG, DHA and HJS critically revised the manuscript. All authors read and approved the final manuscript.
